# Cultivating adaptive expertise in critical care: reimagining intensive care unit education in the era of Artificial Intelligence

**DOI:** 10.62675/2965-2774.20260040

**Published:** 2026-07-22

**Authors:** Miini Minami Teng, Sian Hsiang-Te Tsuei, Maya Koblanski, Leo Anthony Celi

**Affiliations:** 1 The University of British Columbia Vancouver Canada The University of British Columbia - Vancouver Campus - Vancouver, Canada; 2 Massachusetts Institute of Technology Laboratory for Computational Physiology Cambridg Massachusetts United States Laboratory for Computational Physiology, Massachusetts Institute of Technology - Cambridge, Massachusetts, United States

## FROM PREDICTION TO PARTICIPATION: INTENSIVE CARE UNIT TEAM-PATIENT-ARTIFICIAL INTELLIGENCE INTERACTIONS

In the modern intensive care unit (ICU), clinicians meet patients through layers of data and documentation. Artificial Intelligence (AI) is embedded into ICU workflows to predict outcomes, summarize charts, draft handoffs, and triage. When AI participates in sensemaking, harms may appear less as wrong numbers and more as distortions of context: omissions, overconfident summaries, or misframed trends.^(1-3)^

This viewpoint argues that ICU education must evolve beyond fixed AI competencies toward cultivating adaptive expertise. Intensive care unit teams make high-stakes, time-pressured decisions under uncertainty while addressing equity, ethics, and sustainability. Our objective is to outline curriculum reform principles and practical strategies for integrating AI education into ICU workflows.

**Figure 1:**
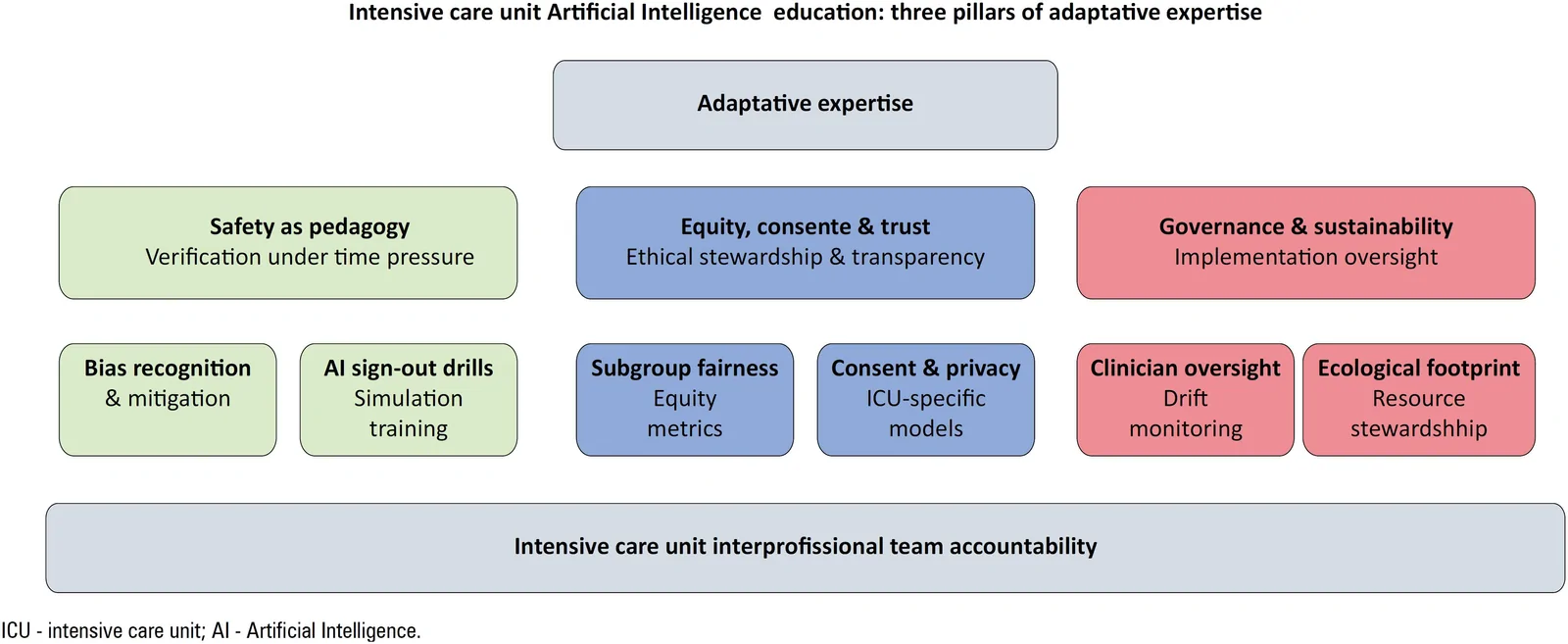
Conceptual framework for intensive care unit Artificial Intelligence education: three pillars anchored in adaptive expertise and unified by interprofessional team accountability.

Medical education has often responded to new technologies by adding fixed competencies. However, in critical care, this approach is insufficient because decisions are time-pressured, high-stakes, and values-laden, often made under physiologic uncertainty and with substitute decision-makers. Intensive care unit curricula should be designed for continual re-evaluation as clinical work and AI systems evolve, preparing teams to adapt. Adaptive expertise – "the flexible use of knowledge and the ability to generate new solutions […] in response to novelty and complexity" – allows teams to recalibrate judgment, ethical reasoning, and professional responsibility as AI reshapes bedside work (Figure 1).^(4,5)^

The figure was created by the authors with the assistance of the Claude Artificial Intelligence tool.

## SAFETY AS PEDAGOGY: TEACHING VERIFICATION UNDER TIME PRESSURE

Foundational AI literacy remains important, but ICU education should emphasize verification skills over technical fluency. Clinicians do not need to code, but must understand common failure modes: bias, hallucinations, omissions, and calibration drift.^(3,4,6)^ For example, in the ICU, the most dangerous failures may be silent: an AI-generated sign-out that sounds coherent yet misses a rising vasopressor requirement, misstates device settings, or compresses uncertainty into false certainty.^(1-3)^

AI biases can shape clinical interpretation even when outputs appear technically correct.^(7)^ Training should explicitly include recognition and mitigation of AI biases alongside verification of outputs. Metrics should include performance inequities across subgroups, calibration by demographic group, and area-under-the-curve comparisons. Training should therefore cover structured critical appraisal of AI outputs - parallel to how evidence-based medicine teaches appraisal of trials - and strategies for staying clinically competent when AI tools are unavailable or untrustworthy.^(4)^

Some humanistic goals, like intellectual humility or AI-independent resilience, are hard to ‘teach’ but can be built through activities that reveal the limits of the tools clinicians already trust. Learners can audit a common ICU score or protocol (e.g., sepsis triggers or Sequential Organ Failure Assessment [SOFA] scores), tracing its development, assumptions, and performance variation across sites.^(1,2)^

Simulation can make verification teachable. In an ‘AI sign-out drill’, a team reviews an AI-generated ICU summary. It must link key claims to source data, identify what is missing, articulate uncertainty, and decide what would trigger a stop-the-line escalation. This treats verification as a team skill aligned with ICU safety culture.

## EQUITY, CONSENT, AND TRUST IN HIGH-ACUITY ENVIRONMENTS

Equity begins with ethical stewardship: how benefits, risks, and accountability are distributed across patients and populations. Intensive care unit AI tools may rely on data streams shaped by structural inequities in access, documentation, and prior care. Without a reflexive lens, ‘objective’ outputs may obscure social drivers of critical illness or amplify disparities through uneven performance across subgroups.^(1,6)^

Equity in critical care is also relational, shaped by how AI-mediated decisions are communicated and experienced in moments of crisis, often with substitute decision-makers and uncertainty. For example, the same AI risk estimate can either build trust or deepen distress depending on whether clinicians present it as a supportive input alongside the patient's values or as a decisive "computer verdict". Disclosure of AI involvement may influence trust and perceived empathy, underscoring the need for transparency paired with careful communication that centers on patient and family priorities.^(8)^

Consent and privacy require special attention in the ICU. Ambient or semi-ambient AI used for documentation, handoffs, or workflow support can extend surveillance beyond traditional charting. Standard consent models often do not fit critical care: patients frequently lack decisional capacity, family presence may be inconsistent, and care is time-sensitive.^(9)^ Intensive care unit curricula should include cases that force learners to navigate these realities: what counts as meaningful consent, how to minimize data exposure, and how to involve patient and family advisors in governance if feasible.

## BUILDING JUST PATHWAYS FOR INTENSIVE CARE UNIT ARTIFICIAL INTELLIGENCE: GOVERNANCE, MONITORING, AND SUSTAINABILITY

Equity also depends on how AI is implemented in clinical workflows. The experience of electronic health records shows that introducing digital tools without meaningful clinician input can disrupt workflow, increase documentation burden, and contribute to burnout.^(10)^ Intensive care unit AI could reproduce these harms - or create new ones by shifting verification labor onto already strained team members. Successful deployment should be defined not only by accuracy but also by improvements in patient-centered outcomes and clinician workload, without degrading safety culture or equity.^(1,2)^

For critical care educators, this means teaching governance as part of professionalism. Trainees should learn to ask deployment-level questions: What is the intended use, and what decisions could this output influence? What evidence supports the benefit in real ICU workflows? Who is accountable when AI content enters the chart or shapes triage? What is the rollback plan when performance drifts?^(2,6)^ A minimum safety bar – prospective evaluation, human oversight, incident learning pathways, and drift monitoring – should be treated as non-negotiable ICU infrastructure.^(1,2,6)^

Finally, ICU AI has material footprints. Training and inference for large models consume energy and water resources, and scaling these tools across health systems can compound emissions and resource demands.^(11,12)^ A critical care lens does not require every clinician to become a sustainability expert. However, curricula should make ecological stewardship a design constraint: learners should justify trade-offs and recognize when simpler approaches are preferable to large, opaque systems.

AI will continue to evolve, and critical care will remain a proving ground for its promises and risks. Reforming ICU education now - toward adaptive expertise, verification under time pressure, and team-based accountability - is essential so tomorrow's ICU clinicians can lead responsibly in a world where clinical work is increasingly AI-mediated.

## Data Availability

The contents are already available.
